# Understanding the scope of practice of physician associate/physician associate comparable professions using the World Health Organization global competency and outcomes framework for universal health coverage

**DOI:** 10.1186/s12960-023-00828-2

**Published:** 2023-06-23

**Authors:** Mary Showstark, Jami Smith, Trenton Honda

**Affiliations:** 1 Yale School of Medicine Physician Assistant Online Program, 100 Church Street South, Suite A230, New Haven, CT 06519 USA; 2 Delaware Valley University Physician Assistant Program, 700 E. Butler Ave., Doylestown, PA 18901 USA; 3 School of Clinical and Rehabilitation Sciences, Northeastern University, 360 Huntington Ave, Boston, MA 02115 USA

**Keywords:** Physician assistant/physician associate, Assistant medical officer, Medical licentiate, Community health officer, Scope of practice, Practice activities, Paramedicine, Clinical officer/clinical associate

## Abstract

**Background:**

Physician Associate and Physician Associate comparable (PA/PA-comparable) professions are classified by the 2012 International Labour Classification of Occupations within ISCO group 2240 paramedical practitioners. However, to date, there is no single global framework which categorizes and/or describes their scopes of practice, or a single unifying occupational group name. In 2022, the World Health Organization (WHO) published its Global Competency and Outcomes Framework for Universal Health Coverage which focuses on the practice activities for health workers with a pre-service training pathway of 12–48 months, thus including many PA/PA-comparable roles. In this study we describe the similarities and differences between the SOP documents for PA/PA-comparable professions with a pre-service pathway of 12–48 months, thus excluding any extra-training and specializations, from 25 countries using the WHO Framework as a frame of reference.

**Methods:**

SOP documents were collected from 25 countries and mapped to the WHO Framework by 3 independent reviewers. We used descriptive statistics to examine the percent agreement between the WHO Framework and SOP documents by country, as well as the ubiquity of each WHO practice activity across the examined documents. To test the hypothesis that country-specific economic indicators and healthcare workforce metrics may be linked to the presence or absence of specific SOP elements, we utilized Wilkoxon and Fisher Exact tests to examine associations between World Bank economic indicators and country specific healthcare workforce metrics and presence/absence of specific WHO Framework practice activities within each SOP.

**Results:**

We identified significant heterogeneity between the WHO practice activities reported in the 25 SOP documents, particularly related to the provision of individual health services. We also identified statistically significant associations between World Bank economic indicators and country specific healthcare workforce metrics and presence/absence of the following seven practice activities relating to Individual Health, Population Health, and Management and Organization practice domains: (1) “Formulating a judgement following a clinical encounter,” (2) “Assessing community health needs” (3) “Planning and delivering community health programmes,” (4) “Managing public health communication,” (5) “Developing preparedness for health emergencies and disasters, including disease outbreaks,” (6)** “**Providing workplace-based learning and supervision,” and (7) “Participating in evaluation and research.” In each case, presence of the above practice activities was associated with lower health economic and workforce indicators, suggesting that these SOP practice activities are more common in lower income countries and countries with a smaller per-capita health workforce.

**Conclusions:**

The WHO practice activities provide an effective framework to catalogue and compare the responsibilities of PA/PA-comparable professions recorded by country specific SOP documents. This approach could also be used to compare different occupational SOPs within a country, as well as SOPs between countries. The authors propose that additional information relating to the types of procedures and the level of supervision or autonomy would enable a more comprehensive comparison of SOPs, going beyond the granularity offered by the WHO framework. At that level, the evaluation could then be used to inform gap analyses for training needs in the context of migration, or to better understand the health team skill mixes across different countries. The study also offers reflections on the importance of clarity of intended meaning within the SOP documents.

## Key points

- Describe the World Health Organization Global Competency and Outcomes Framework for Universal Health Coverage.
- Compare the Scope of Practice of PAs and comparable professions using the WHO Global Competency and Outcomes Framework for Universal Health Coverage.
- Identify similarities and differences in the practice activities among PAs and comparable professions as it relates to geography, health expenditure, and wider health workforce composition.
- Evaluate the utility of the practice activities in the Global Competency and Outcomes Framework as a mechanism to describe and compare Scopes of Practice.

## Background

Many countries’ health workforce includes medically trained health workers who do not identify as physicians or nurses; yet are known to take on many similar diagnostic and clinical functions as physicians. Various terminology has been proposed to describe to this group of health workers; for the purposes of this paper we utilize the term Physician Associate/Physician Associate comparable (PA/PA-comparable) [1–5].

PA/PA-comparable professions exist in over 50 countries under a variety of different titles [6] (Table 1), with varying models for education, training, specialization, regulation, and accreditation [8, 9]. Generally, the scope of practice (SOP) for the PA/PA-comparable professions are delineated in SOP documents. A SOP defines what a health worker is allowed to do, as defined by their educational competencies, regulatory bodies, government and/or their workplace statutes [10]. The SOP offers a single reference for health workers, employers, education institutions, patients, and communities alike. Effective team functioning requires clear understanding of one’s own SOP, as well as that of other health team members, and enables a clear delineation of roles and responsibilities in patient care. Further, a clear SOP enables curriculum outcomes to be defined and assessed consistently, and informs workforce planning.

**Table 1:** Physician associate and physician associate-comparable profession titles of the SOP documents reviewed in this study

Titles of the profession	Countries
Assistant Medical Officer	Malaysia
Clinical Associate	South Africa
Clinical Officer	Burundi, Kenya, Malawi, Rwanda, Uganda, Zambia*
Community Health Officer	Sierra Leone
Emergency Surgical/Pediatric and Child Health/ Mental Health Officer	Ethiopia*
Health Officer	Ethiopia*, Zimbabwe
Medical Assistant	Bangladesh
Medical Licentiate	Zambia*
Physician assistant	Australia, Canada, Germany, Ghana, India, Israel, Liberia, Netherlands, United States*
Physician associate	Ireland, Switzerland, New Zealand, United Kingdom, United States*

*Three SOP documents related to PA/PA-Comparable roles with more than one job title commonly used within the country. For clarity both job titles are included in this table, although only a single SOP was identified

PA/PA-comparable professions are classified by the 2012 International Labor Organization International Standard Classification of Occupations (ISCO-08) within Group 2240: Paramedical Practitioners [7]. ISCO group 2240 is defined as “paramedical practitioners {who} provide advisory, diagnostic, curative and preventive medical services more limited in scope and complexity than those carried out by medical doctors. They work autonomously, or with limited supervision of medical doctors, and apply advanced clinical procedures for treating and preventing diseases, injuries and other physical or mental impairments common to specific communities.” This thus also includes medically trained health workers with a narrow SOP such as primary care paramedics (first responders) or surgical technicians (who assist a medical doctor during procedures). The PA/PA-comparable group self-identify with a broader range of responsibilities, including the provision of individual healthcare, population health, and healthcare management and organization, although the nature and extent of these vary by setting of practice and the health workforce team in those settings.

In 2022, the World Health Organization (WHO) published its Global Competency and Outcomes Framework for Universal Health Coverage [9], hereafter referred to as the WHO Framework. The WHO Framework provides guidance for developing competency-based education programmes, oriented towards programmes of 12–48 months’ duration, thus directly inclusive of PA/PA-comparable roles. Whilst there are a number of existing cross-cutting frameworks on specific aspects of practice, as well as several occupation-specific frameworks, this is the first outcomes framework that is explicitly inclusive of the PA/PA-comparable profession. The WHO Framework differentiates between the functional outcomes of a health worker training programme (which reflect the activities and tasks typically found in job descriptions or used to allocate responsibilities within a team, referred to as practice activities), and the behavioural outcomes of a programme (which are the standards for the performance of the practice activities, referred to as competencies). Practice activities are the core functions of health practice. They comprise groups of related tasks that may be undertaken by one person or groups of people. It is thus the practice activities, rather than the competencies, that are relevant for comparison of PA/PA-comparable professions’ SOPs.

The WHO Framework identifies 35 practice activities, noting that typically, a single role or person would not have responsibilities across them all. The practice activities have been developed through the lens of educational outcomes, and thus emphasize a common knowledge and skills base, as well as the broader role responsibilities. This includes practice activities that are not typically regulated by scopes of practice, for example care coordination, team handovers, and delivering quality improvement activities. The full list of practice activities (Table 2) are organised into three domains: “individual health” (relating to the provision of health services for an individual), “population health” (relating to the provision of health services for communities) and “management and organization” (relating to the effective use of human, physical and financial resources) [9].

**Table 2:** WHO Global Competency and Outcomes Framework for UHC: practice activities [9] plus three sub-categories added for this study

Domain	No.	Title	Description
Domain I: Individual Health Domain Practice activities relating to the provision of health services for an individual	1.	Gathering information through interviewing and assessment	Tasks within this practice activity relate to gathering and confirming information through interviewing, taking a history and performing a cognitive, emotional, mental, physical or social assessment (NB excluding diagnostic testing)
	2.	Formulating a judgement following a clinical encounter	Tasks within this activity relate to interpreting information and clinical decision-making; this is the process of making a diagnosis or classifying a condition or action plan, for example, based on a decision-making tree
	3.	Managing conversations with individuals and their families	Tasks within this activity may include difficult conversations, such as discussing a diagnosis, prognosis or management plan. This is an overarching outcome relating to “managing the patient encounter” and is relevant to many other practice activities such as gathering information or developing the treatment plan; but it would duplicate the resource mapping
	4.	Advocacy for individual health needs	Tasks within this practice activity include supporting the individual to access health services; this may include practical aspects of help, helping the individual increase their health literacy or representing the individual in decision-making about their care
	5.	Providing information and support to impact individual health behaviours	Tasks towards this practice activity relate to health promotion and prevention goals of care; such as providing information about positive or harmful behaviours, distributing non-medical supplies such as bed nets, or monitoring and tracking behaviour change. Tasks within this practice activity may follow from an individual’s diagnosis and managing their health risks and so they are tailored to that individual, but they exclude post-procedural care or post-procedural counselling
	6.	Gaining informed consent	Tasks within this practice activity comprise the component steps of sharing or clarifying information, addressing concerns, and confirming and documenting consent
	7.	Ordering, administering and interpreting the results of diagnostic and screening procedures	Tasks within this practice activity relate to determining the need for a test or procedure, carrying that procedure out, and interpreting the results of the test. NB This is distinct from using those results to determine the implications of the results towards developing a treatment and management plan, in Practice Activity 8
	8.	Developing and adjusting a management plan	Tasks within this practice activity are part of the process to interpret information about the individual and their health needs, and to determine with them the management plan. Tasks also reflect the process of implementing the management plan, including identifying resources and coordinating others’ roles; as well as interpreting monitoring information and revising and adjusting a plan, including the withdrawal or end of a plan
	9.	Prescribing medications or therapeutics	Tasks within this practice activity reflect the decision-making process towards prescribing decisions, as well as the documentation of a prescription, and taking actions to ensure the follow-up or monitoring of the medications within a wider management plan
	10.	Preparing and dispensing medications or therapeutics	Tasks within this practice activity describe any preparation or mixing of medications prior to dispensing to the individual; and the interaction of dispensing including providing information about self-administration and side-effects
	11.	Administering medications or therapeutics	Tasks within this practice activity include any preparation for administration, the administration of medications/therapeutics, as well as monitoring for side-effects. NB This excludes self-administration; this describes the administration of a medication by the health workers
	12.	Selecting assistive products	Tasks within this practice activity include the specific assessments to select and specify an assistive product, ordering the product and writing instructions for its use
	13.	Providing assistive products	Tasks within this practice activity are the preparing, fitting and/or setting up of the product, as well as supporting the individual to use it. Tasks also include monitoring and follow-up, and basic maintenance and repairs or adaptations of the product
	14.	Providing non-pharmacological health interventions	This includes all tasks (excluding medications or therapeutics) towards a single intervention, procedure or health service: this includes preparing the individual for the procedure; performing or assisting in the provision of a procedure; monitoring the individual’s response during or following the procedure including follow-up visits; and post-procedure counselling
	15.	Providing treatment and care support to individuals	Tasks within this practice activity are related to the individual, rather than the provision of a procedure. This might include clinical support tasks such as wound care, often in a different setting to the preceding non-pharmacological interventions, e.g. surgery; or it might be education and training the individual in self-care; or providing emotional or psychological support for coping with a diagnosis or steps in a management plan
	15a	Providing for an individual Emergency response	Additional sub-category
	16.	Managing end-of-life and bereavement care	Tasks within this practice activity are specifically oriented towards end of life, including provision of palliative care, providing comfort and psychosocial support to the individual and their carers. It excludes signing a death certificate (part of Practice Activity 27: documentation)
	16a	Providing a death declaration	Additional sub-category
	16b	Managing hospice care	Additional sub-category
	17.	Reporting notifiable diseases, conditions or events	Tasks within this practice activity relate to reporting and surveillance of an individual’s health status (disease, condition or event) in relation to reporting criteria
	18.	Providing a clinical presentation to other health workers	Tasks within this practice activity encompass handovers and referrals for the purpose of transferring responsibility for care; it could take verbal or written form
	19.	Moving and transporting individuals	Tasks within this practice activity describe the moving, handling and transporting of individuals, including planning, preparation of aids, monitoring the individual during the episode and returning equipment for subsequent use
	20.	Coordinating transfer to another care environment	All practical tasks towards the transfer of care between environments—determining the basis for the transfer is part of Practice Activity 8: developing and adjusting a management plan. Tasks here include evaluating options, developing a transfer plan, making practical arrangements and care coordination
Domain II: Population Health Domain Practice activities relating to the provision of health services for communities	21.	Assessing community health needs	Tasks within this practice activity relate to efforts to plan for an assessment, coordinate governance, collect and interpret information, propose options to address the findings, and report on the findings
	22.	Planning and delivering community health programmes	Tasks within this practice activity are acting on the findings of a community health assessment, from planning a programme, preparing, implementing and evaluating the programme
	23.	Managing public health communication	Tasks within this practice activity relate to public (mass) communications, from determining the need for communications, selecting the appropriate method, developing communications materials and content and evaluating the communications efforts
	24.	Developing preparedness for health emergencies and disasters, including disease outbreaks	Tasks within this practice activity are around strengthening community preparedness in the case of an emergency or disaster, from assessing preparedness, participating in, planning or running preparedness activities, preparing resources, monitoring and evaluating preparedness, and monitoring and evaluating the risks of health emergencies and disasters according to health data
	25.	Responding to health emergencies and disasters, including disease outbreaks	Tasks within this practice activity describe the efforts to respond to known emergencies or disaster events, including monitoring the situation, ensuring communications, organizing supplies and resources, monitoring, evaluating and coordinating the response, and developing recovery plans. NB This relates to the response as a collective effort but excludes tasks towards individual health services which are mapped to practice activities 1–20 in the Individual Health Domain
	26.	Advocacy for community health needs	Tasks within this practice activity include the breadth of actions to advocate for and with a community, from developing an advocacy strategy, identifying stakeholders, mobilizing the community, taking actions and evaluating the impact
	27.	Accessing and documenting information	Tasks towards accessing, documenting and validating information, including issuing legal documentation such as prescriptions or death certifications. More broadly, this practice activity also describes tasks towards developing information management structures
Domain III: Management and Organization Domain Practice activities relating to the effective use of human, physical and financial resources	28.	Registering individuals for health services	Tasks within this practice activity include outreach to identify the individuals to be registered (for example, contact tracing), as well as conducting initial (non-clinical) triage, gathering information and inputting that information into a health information system
	29.	Delivering quality improvement activities	Tasks within this practice activity include identifying areas of improvement, planning, implementing, coordinating, overseeing and evaluating impact of quality improvement initiatives
	30.	Providing workplace-based learning and supervision	Tasks within this practice activity are those towards providing supervision, feedback, formal or informal workplace-based learning, and reporting on performance. NB Beyond the pre-service training pathway for health workers for 12–48 months (the lens through which this framework has been developed), this could be extended to include specialized education responsibilities including curricula design, development and implementation, assessment or standard-setting and developing curricula resources
	31.	Managing human resources	Tasks within this practice activity are those towards coordinating, overseeing and planning for the performance of other health workers, such as performance management, estimating workforce needs, scheduling or taking actions to ensure safety and well-being in the workplace
	32.	Managing financial resources	Tasks within this practice activity include managing a budget, making budget decisions, keeping financial records or processing payments or billing
	33.	Managing physical resources	Tasks within this practice activity relate to the use of, storing or maintaining physical resources such as equipment or workspaces. This also includes making decisions around the use of physical resources and stock control
	34.	Participating in evaluation and research	Tasks within this practice activity relate to sharing, collecting or recording information as it relates to research activities, as well as interpreting findings. NB Beyond the pre-service training pathway for health workers for 12–48 months (the lens through which this framework has been developed), this could be extended to include specialized research responsibilities including ethical approvals, research study design, conducting research, writing and disseminating findings
	35.	Developing, evaluating and implementing local policies, procedures and guidelines	Tasks within this practice activity incorporate contributions to a collective policy-making process, from contributing information, gathering data, evaluating information, drafting text or translating a policy into local procedures; as well as piloting or monitoring implementation

Purpose of Article: The aim of this exploratory study is to describe the similarities and differences between the SOP documents for PA/PA-comparable professions from 25 countries using the practice activities of the WHO Global Competency and Outcomes Framework for UHC as a frame of reference. Utilizing the WHO Framework, we assess the heterogeneity of PA/PA-comparable SOP documents from 25 countries and examine whether presence or absence of specific activities within SOPs is linked to country-specific healthcare expenditure and workforce metrics.

## Methods

A request for SOP documentation was made to a global community of PA/PA-comparable professions through the International Academy of PA Educators, International PA Organization, Global Association of Clinical Officers and Physician Assistants (see appendix 1). Scope of Practice (or comparable) documents were collected from twenty-five countries in 2020 comprising twelve countries in Africa, six in Asia/Oceania, five in Europe, and two in North America. Four SOP documents were provided in languages other than English (Bengali, Dutch, German and French) and were translated to English prior to evaluation using Google Translate. Where more than one PA/PA-comparable professional SOP was identified within a single country document, the SOP relating to a 48-month training program, rather than a specialized PA/PA-comparable profession, was selected. The SOP documents were primarily sourced from governmental entities, however where these were unavailable, others were obtained from educational institutions, national advocacy organizations, and other sources (Table 3).

**Table 3:** Type of documentation provided by each country

Source of scope of practice documents	Number of countries	Countries
Official document from a government entity (i.e. Ministry of Health or others)	13	Australia, Ethiopia, Germany, Ghana, Ireland, Kenya, Malawi, Malaysia, Rwanda, South Africa, Uganda, Zambia, Zimbabwe
Obtained from national PA organization	6	Bangladesh, Canada, India, the Netherlands, United Kingdom, United States
Obtained from an educational program	3	Burundi, Liberia, Sierra Leone
Other (concept paper, description from practicing PA in country, or websites)	3	Israel, New Zealand, Switzerland

Each SOP document was reviewed to identify the presence or absence of tasks pertaining to each of the 35 WHO practice activities (and 2 sub-categories). The detail recorded within the SOP did not always differentiate between knowledge of and performance of individual practice activities (e.g., knowledge of prescribing is not the same as prescribing). As such, during data extraction only the practice activities that were explicitly stated as performed were mapped. This process was completed by two reviewers and quality checked by a third reviewer. As presence of a SOP element was coded as 1 and absence was coded as 0, there were no missing values in the dataset.

While the WHO Framework identifies 35 practice activities, we added two sub-categories to practice activity number “16. Managing end-of-life and bereavement care” as follows: “16a: Providing a death declaration”, and “16b: providing hospice care”. This was done to better understand the role of PA/PA-comparable professions around end-of-life care. Likewise, during the initial data extraction practice activity 15. Providing treatment and care support to individuals was expanded with a sub-category to capture the provision of emergency response care (15a. Providing for an individual Emergency Response). The final list of 35 Practice Activities and three sub-categories are shown in Table 2. Data were summarized using descriptive statistics. Wilcoxon test for independent samples (for continuous predictor variables) and Fisher exact tests (for binary predictor variables) were used to examine associations between the presence/absence of a WHO practice activity within the SOP, and variables that we believed a priori would potentially be predictors of SOP elements, including: Health expenditure per capita (continuous), medical doctors per 1000 population (continuous), and nurses per 1000 population (continuous). Additionally, differences in presence/absence of practice activities according to the World Bank designation of High versus Low/Middle income countries was assessed (binary). All statistical analyses were conducted with R 3.6.1. (R Core Team, 2019).

## Results

Scope of Practice documents were collected from twenty-five countries in 2020 comprising twelve countries in Africa, six in Asia/Oceania, five in Europe, and two in North America. Table 4 shows the percent of WHO practice activities represented in SOP documents for each country. The percentage of WHO Framework practice activities identified ranged from 15.8% (Israel) to 73.7% (Zambia). Overall, the SOP documents from the Africa region contained the largest percent of WHO practice activities, with the regional average being 57.7%. Regional averages for Southeast Asia, Europe, and the Americas were all appreciably lower, at 32.9%, 49.5%, and 48.7%, respectively.

**Table 4:** Percent of WHO practice activities represented in country SOP documents ^a^

Country	Percent agreement
*Africa*	
– Burundi	63.2
– Ethiopia	63.2
– Ghana	55.3
– Kenya	60.5
– Liberia	47.4
– Malawi	52.6
– Rwanda	44.7
– Sierra Leone	71.1
– South Africa	36.8
– Uganda	63.2
– Zambia	73.7
– Zimbabwe	60.5
*Asia/Oceania*	
– Australia	50.0
– Bangladesh	39.5
– India	23.7
– Malaysia	39.5
– New Zealand	28.9
*Europe*	
– Germany	50.0
– Ireland	60.5
– Netherlands	44.7
– Switzerland	42.1
– United Kingdom	50.0
– Israel	15.8
*North America*	
– Canada	50.0
– United States	47.4

The percentage of SOP documents that contained the individual WHO practice activities varied by domain. For example, the practice activities within the domain of “Individual Health” ranged from 12 to 100% in the SOP documents as demonstrated in Table 5. Ranges were similar for the “Management and Organization” domain (8–92%), while the range was somewhat smaller for the “Population Health” domain (20–48%). Additionally, within the “Individual Health” domain, 48% of practice activities were represented in more than 75% of country SOP documents while for the “Population Health” domain and “Management and Organization” domain, 0% and 11% were represented in greater than 75% of country SOP documents respectively.

**Table 5:** Number and percent of country SOP documents containing tasks mapped to WHO practice activities and 3 sub-categories

WHO practice activities	Number (%) of Country SOPs with WHO practice activity
*Individual health*	
1. Gathering information through interviewing, examination and assessment	1 (88%)
2.Formulating a judgement following a clinical encounter	25 (100%)
3.Managing conversations with individuals and their families	21 (84%)
4.Advocacy for individual health needs	18 (72%)
5.Providing information and support to impact individual health behaviours	21 (84%)
6.Gaining informed consent	9 (36%)
7.Ordering, administering and interpreting the results of diagnostic and screening procedures	22 (92%)
8.Developing and adjusting a management plan	21 (84%)
9.Prescribing medications or therapeutics	10 (44%)
10.Preparing and dispensing medications or therapeutics	2 (12%)
11.Administering medications or therapeutics	14 (56%)
12.Selecting assistive products	3 (12%)
13.Providing assistive products	6 (24%)
14.Providing non-pharmacological health interventions	25 (100%)
15.Providing treatment and care support to individuals	23 (92%)
15a. Providing for an individual Emergency Response	25 (100%)
16.Managing end-of-life and bereavement care	0 (0%)
16a. Providing a death declaration	3 (12%)
16b. Managing hospice care	1 (0%)
17.Reporting notifiable diseases, conditions or events	6 (24%)
18.Providing or receiving a clinical presentation	19 (80%)
19.Moving and transporting individuals	4 (12%)
20.Coordinating transfer to another care environment	15 (60%)
*Population health*	
21.Assessing community health needs	11 (40%)
22.Planning and delivering community health programmes	12 (48%)
23.Managing public health communication	5 (20%)
24.Developing preparedness for health emergencies and disasters, including disease outbreaks	6 (24%)
25.Responding to health emergencies and disasters, including disease outbreaks	7 (32%)
26.Advocacy for community health needs	7 (28%)
*Management and organization*	
27.Accessing and documenting information	22 (92%)
28.Registering individuals for health services	4 (16%)
29.Delivering quality improvement activities	15 (64%)
30.Providing workplace-based learning and supervision	13 (52%)
31.Managing human resources	8 (36%)
32.Managing financial resources	7 (28%)
33.Managing physical resources	14 (56%)
34.Participating in evaluation and research	16 (64%)
35.Developing policies and protocols	2 (8%)

The analysis of the WHO practice activities and selected country-level healthcare resource metrics highlighted significant associations for six practice activities. The presence/absence of one practice activity in the ‘Individual Health’ domain [2) “Formulating a judgement following a clinical encounter,”] was significantly associated with medical doctors per 1000 population (Absent median < 0.00, present median 1.35, *Z* = 9, *p* = 0.05) and nurses per 1000 population (absent median 0.40, present median 3.85, *Z* = 1.5, *p* = 0.01) (Table 6).

**Table 6:** Associations between the presence of Individual Health WHO practice activities in country SOP documents and country-level healthcare resource metrics

WHO practice activities	SOP element present	Health expenditures per capita^a^		Medical doctors per 1000 population^a^		Nurses per 1000 population^a^		High vs. low income^a,b^
		N (Median)	Statistic^c^ (*p*-value)	N (Median)	Statistic^c^ (*p*-value)	N (Median)	Statistic^c^ (*p*-value)	OR^d^ (*p*-value)
1.Gathering information through interviewing, examination and assessment	Absent:	16 (229)	60 (0.52)	16 (0.75)	70 (0.93)	16 (1.50)	53 (0.29)	2.63 (0.39)
	Present:	9 (4338)		9 (2.60)		9 (8.20)		
2.Formulating a judgement following a clinical encounter	Absent:	3 (105)	13 (0.11)	3 (< 0.00)	**9** (0.05)*	3 (0.40)	**1.5** (0.01)*	‡
	Present:	22 (1118)		22 (1.35)		22 3.85)		
3.Managing conversations with individuals and their families	Absent:	0 (‡)	‡	0 (‡)	‡	0 (‡)	‡	‡
	Present:	25 (253)		25 (0.90)		25 1.90)		
4.Advocacy for individual health needs	Absent:	4 (1634)	48 (0.70)	4 (2.25)	50 (0.58)	4 (3.70)	39 (0.85)	0.63 (1.00)
	Present:	21 (201)		21 (0.90)		21 1.90)		
5.Providing information and support to impact individual health behaviours	Absent:	7 (1139)	79 (0.36)	7 (1.50)	75 (0.49)	7 (4.20)	82 (0.26)	0.85 (1.00)
	Present:	18 (203)		18 (0.75)		18 1.30)		
6.Gaining informed consent	Absent:	4 (2010)	48 (0.70)	4 (2.25)	48 (0.68)	4 (7.05)	46.5 (0.77)	0.63 (1.00)
	Present:	21 (205)		21 (0.90)		21 1.90)		
7.Ordering, administering and interpreting the results of diagnostic and screening procedures	Absent:	2 (617)	‡	2 (1.05)	23 (1.00)	2 (1.95)	13.5 (0.37)	‡
	Present:	23 (253)		23 (0.90)		23 1.90)		
8.Developing and adjusting a management plan	Absent:	4 (194)	32 (0.50)	4 (0.55)	42 (1.00)	4 (1.45)	34 (0.58)	2.18 (0.63)
	Present:	21 (1098)		21 (1.20)		21 3.50)		
9.Prescribing medications or therapeutics	Absent:	14 (696)	82 (0.81)	14 (1.35)	93.5 (0.38)	14 (3.85)	96.5 (0.30)	0.77 (1.00)
	Present:	11 (205)		11 (0.60)		11 (1.30)		
10.Preparing and dispensing medications or therapeutics	Absent:	22 (229)	23 (0.45)	22 (0.90)	25.5 (0.56)	22 (1.50)	12 (0.09)	3.32 (0.54)
	Present:	3 (4816)		3 (3.3)		3 (12.60)		
11.Administering medications or therapeutics	Absent:	11 (1098)	80 (0.89)	11 (0.90)	87.5 (0.58)	11 (1.70)	71 (0.76)	1.30 (1.00)
	Present:	14 (203)		14 (0.70)		14 3.05)		
12.Selecting assistive products	Absent:	22 (229)	25 (0.55)	22 (1.05)	31.5 (0.93)	22 (2.60)	24.5 (0.50)	0.73 (1.00)
	Present:	3 (1098)		3 (0.90)		3 (1.90)		
13.Providing assistive products	Absent:	19 (253)	52 (0.78)	19 (0.90)	61 (0.82)	19 (1.70)	51.5 (0.75)	1.67 (0.65)
	Present:	6 (2270)		6 (1.40)		6 (5.05)		
14.Providing non-pharmacological health interventions	Absent:	0(‡)	‡	0 (‡)	‡	0 (‡)	‡	‡
	Present:	25 (253)		25 (0.9)		25 1.90)		
15.Providing treatment and care support to individuals	Absent:	2 (2056)	27 (0.73)	2 (2.75)	34.5 (0.27)	2 (3.50)	23.5 (1.00)	0.65 (1.00)
	Present:	23 (205)		23 (0.90)		23 1.90)		
16.Managing end-of-life and bereavement care	Absent:	25 (253)	‡	25 (0.90)	‡	25 (1.90)	‡	‡
	Present:	0 (‡)		0 (‡)		0 (‡)		
16a. Providing a death declaration	Absent:	22 (676)	28 (0.72)	22 (1.05)	41 (0.53)	22 (1.50)	21 (0.34)	0.73 (1.00)
	Present:	3 (201)		3 (0.20)		3 (4.20)		
16b. Managing hospice care	Absent:	25 (253)	‡	25 (0.90)	‡	25 (1.90)	‡	‡
	Present:	0 (‡)		0 (‡)		1 (‡)		
17.Reporting notifiable diseases, conditions or events	Absent:	19 (1139)	62 (0.78)	19 (1.50)	75.5 (0.25)	19 (4.20)	71.5 (0.37)	0.73 (1.00)
	Present:	6 (191)		6 (0.20)		6 (1.25)		
18.Providing or receiving a clinical presentation	Absent:	5 (105)	24 (0.08)	5 (0.60)	41 (0.56)	5 (0.70)	23 (0.07)	3.13 (0.61)
	Present:	20 (696)		20 (1.35)		20 3.85)		
19.Moving and transporting individuals	Absent:	10 (696)	66 (0.64)	10 (1.20)	79 (0.85)	10 (2.60)	66.5 (0.66)	1.00 (1.00)
	Present:	15 (205)		15 (0.90)		15 1.90)		
20.Coordinating transfer to another care environment	Absent:	22 (229)	34 (0.97)	22 (0.90)	26.5 (0.61)	22 (1.80)	28 (0.71)	3.32 (0.54)
	Present:	3 (4338)		3 (2.80)		3 (8.20)		

Bold demonstrate significantly significant results

^a^Per 1000 USD increment

^b^As defined by the World Bank

^c^Wilkoxon test for independent samples

^d^ Fisher exact test

^*^p-value < 0.05

^‡^Data unavailable for these data points

Significant associations were also present for four WHO practice activities in the “Population Health” domain, including “21: Assessing community health needs”, with presence associated with lower health expenditures per capita (Absent median: 3015, present median: 146, *p* = 0.02), fewer medical doctors per 1000 population (Absent median: 2.6, present median 0.10, *p* = 0.02), fewer nurses per 1000 population (Absent median: 5.70, present median: 1.05, *p* = 0.01), and the country being classified by the World Bank as high Income was associated with very low odds of practice activity presence in the SOP (OR 0.01, *p* = 0.05) (Table 7.). Similar significant associations were also identified for “22: Planning and delivering community health programmes” whose presence was associated with lower health expenditures per capita (Absent median: 3015, present median: 146, *p* = 0.03), fewer medical doctors per 1000 population (Absent median: 2.60, present median: 0.15, *p* = 0.03), and fewer nurses per 1000 population (Absent median: 5.70, present median: 1.20, *p* = 0.02). Similar significant associations were observed for, “23. Managing public health communication” [Health expenditures per capita (Absent median: 3768, present median: 153, *p* = 0.01); Medical doctors per 1000 population (Absent median: 2.60, present median: 0.15, *p* = 0.01); Nurses per 1000 population (Absent median: 9.90, present median: 1.20, *p* = 0.02); and high versus low-income country (OR 0.08, *p* = 0.01)] and for “24. Developing preparedness for health emergencies and disasters, including disease outbreaks” [Health expenditures per capita (Absent median: 4338, present median: 141, *p* < 0.01); Medical doctors per 1000 population (Absent median: 2.80, present median: 0.15, *p* < 0.01); Nurses per 1000 population (Absent median: 9.90, present median: 1.20, *p* = 0.01); and high versus low income country (OR 0.04, *p* < 0.01)].

**Table 7:** Associations between the presence of Population Health WHO practice activities in country SOP documents and country-level healthcare resource metrics

WHO practice activities	SOP element present	Health expenditures per capita^a^		Medical doctors per 1000 population^a^		Nurses per 1000 population^a^		High vs. low income^a,b^
		N (Median)	Statistic^c^ (*p*-value)	N (Median)	Statistic^c^ (*p*-value)	N (Median)	Statistic^c^ (*p*-value)	OR^d^ (*p*-value)
21. Assessing community health needs	Absent:	19 (3015)	**94** (0.02)*	19 (2.6)	**93.5** (0.02)*	19 (5.70)	**96.5** (0.01)*	**0.01** (0.05) *
	Present:	6 (146)		6 (0.10)		6 (1.05)		
22. Planning and delivering community health programmes	Absent:	17 (3015)	**105** (0.03)*	17 (2.60)	**105** (0.03)*	17 (5.70)	**108** (0.02)*	0.14 (0.09)
	Present:	8 (146)		8 (0.15)		7 (1.20)		
23. Managing public health communication	Absent:	15 (3768)	**120** (0.01)*	15 (2.60)	**120** (0.01) *	15 (9.90)	**116** (0.02)*	**0.08** (0.01)*
	Present:	10 (153)		10 (0.15)		10 1.20)		
24. Developing preparedness for health emergencies and disasters, including disease outbreaks	Absent:	13 (4338)	135 (< 0.01)*	13 (2.80)	**131** (< 0.01)*	13 (9.90)	**128** (0.01)*	**0.04** (< 0.01)*
	Present:	12 (141)		12 (0.15)		12 1.20)		
25. Responding to health emergencies and disasters, including disease outbreaks	Absent:	20 (2056)	66 (0.30)	20 (1.75)	76.5 (0.08)	20 (4.95)	79 (0.06)	‡
	Present:	5 (180)		5 (0.10)		5 (1.20)		
26. Advocacy for community health needs	Absent:	18 (676)	59 (0.84)	18 (0.90)	64 (0.98)	18 (2.70)	75.5 (0.47)	1.17 (1.00)
	Present:	7 (205)		7 (1.20)		7 (1.30)		

Bold demonstrate significantly significant results

^a^Per 1000 USD increment

^b^As defined by the World Bank

^c^Wilkoxon for independent samples

^d^Fisher exact test

^*^p-value < 0.05

^‡^Data unavailable for these data points

Table 8 shows the associations for the “Management and Organization” WHO practice activities and measures of country-level healthcare resource metrics. Two practice activities (30. Providing workplace-based learning and supervision; 34. Participating in evaluation and research) were associated with Health Expenditures per capita and medical doctors per 1000 population. As seen in the other domains, presence of these practice activities was associated with significantly lower country-level healthcare resource metrics, while high income countries were associated with significantly lower odds of “34. Participating in evaluation and research” being present in the SOP.

**Table 8:** Associations between the presence of Management & Organization WHO practice activities in country SOP documents and country-level healthcare resource metrics

WHO practice activities	SOP element present	Health expenditures per capita^a^		Medical doctors per 1000 population^a^		Nurses per 1000 population^a^		High vs. low income^a,b^
		N (Median)	Statistic^c^ (*p*-value)	N (Median)	Statistic^c^ (*p*-value)	N (Median)	Statistic^c^ (*p*-value)	OR^d^ (*p*-value)
27. Accessing and documenting information	Absent:	2 (1575)	20 (0.81)	2 (2.35)	27.5 (0.69)	2 (3.45)	21 (0.88)	0.65 (1.00)
	Present:	23 (253)		23 (0.90)		23 (1.90)		
28. Registering individuals for health services	Absent:	21 (253)	42 (1.00)	21 (1.20)	45.5 (0.82)	21 (3.50)	58 (0.25)	0.46 (0.63)
	Present:	4 (652)		4 (0.75)		4 (0.85)		
29. Delivering quality improvement activities	Absent:	9 (1098)	77 (0.803)	9 (0.90)	79.5 (0.69)	9 (1.70)	73.5 (0.96)	0.76 (1.00)
	Present:	16 (203)		16 (0.70)		16 (2.70)		
30. Providing workplace-based learning and supervision	Absent:	12 (3391)	**116** (0.04)*	12 (2.70)	**128** (0.01)*	12 (6.95)	113 (0.06)	0.23 (0.11)
	Present:	13 (158)		13 (0.20)		13 (1.20)		
31. Managing human resources	Absent:	9 (3768)	99 (0.14)	9 (2.60)	97 (0.16)	9 (8.20)	104. (0.08)	0.18 (0.09)
	Present:	16 (191)		16 (0.40)		16 (1.25)		
32. Managing financial resources	Absent:	18 (1118)	70 (0.70)	18 (1.20)	79.5 (0.33)	18 (3.85)	76.5 (0.43)	0.51 (0.66)
	Present:	7 (180)		7 (0.20)		7 (1.20)		
33. Managing physical resources	Absent:	16 (2077)	94 (0.23)	16 (2.05)	100 (0.12)	16 (4.95)	98.5 (0.14)	0.30 (0.23)
	Present:	9 (158)		9 (0.20)		9 (1.20)		
34. Participating in evaluation and research	Absent:	11 (3768)	**120** (0.02)*	11 (2.60)	**126** (0.01)*	11 (8.20)	111 (0.07)	**0.17** (0.05) *
	Present:	14 (153)		14 (0.15)		14 (1.20)		
35. Developing policies and protocols	Absent:	23 (1098)	38 (0.17)	23 (1.20)	34.5 (0.27)	23 (3.50)	36 (0.21)	‡
	Present:	2 (112)		2 (0.15)		2 (0.95)		

Bold demonstrate significantly significant results

^a^Per 1000 USD increment

^b^As defined by the World Bank

^c^Wilkoxon for independent samples

^d^Fisher exact test

^*^p-value < 0.05

^‡^Data unavailable for these data points

## Discussion

Ours is the first study to map a cohort of international PA/PA-comparable SOP documents with the WHO Framework. Using the WHO practice activities, we were able to explore the heterogeneity in SOP documents across various countries and geographic regions. We determined that the vast majority of SOP documents included most (and in some cases all) of the WHO Framework elements for “Individual health”. Interestingly, presence or absence of the “Individual Health” practice activities was largely independent of economic indicators in our statistical analyses. While “Population Health” and “Management and Organization” practice activities were less prevalent across the entirety of our sample, many of these practice activities were significantly and inversely associated with country-specific economic indicators. This suggests that in countries and regions with lower economic and healthcare resources, the SOP of the PA/PA-comparable profession reflects a role with a broader range of responsibilities than just provision of ‘Individual Health’ services.

### The WHO Framework and its practice activities

We determined that the WHO Framework comprehensively describes the breadth of practice activities found within the PA/PA-comparable professions’ SOP documents. This is evidenced by all practice activities in the SOP documents mapping readily to the WHO Framework.

However, the WHO Framework did not fully capture the depth and complexity of the practice activities found within all the SOP documents across our sample. For example, certain SOP documents had detailed prescribing and administering medication lists for the PA/PA-comparable to utilize. Consistent with this, specific procedures—ranging from suturing to caesarian sections—vary between SOP documents, but this granularity is not captured in the WHO Framework.

The most prevalent practice activities across all SOP documents were from the ‘Individual Health’ domain. This may reflect that the role of the PA/PA-comparable profession is typically developed in response to the need for direct patient care. However, there were significant differences observed within the ‘Individual Health’ domain across countries. For example, 100% of the SOP documents reflected that PA/PA-comparable professions may provide “14: (…) non-pharmacological health interventions” including procedures, however only 36% included “6: gaining informed consent” within the SOP document. This may result from country-specific differences in implied standard of care, such that in some countries it would be assumed that informed consent would be obtained from a patient before performing a procedure, while in others this was explicitly stated within their SOP document.

Of note, not all practice activities were found in all SOP documents. This consistent with the WHO Framework encompassing the practice activities of numerous professions. For example, “10: Preparing and dispensing medications or therapeutics” may be more likely to be related to the role of the nurse professional, and “19: Moving and transporting individuals” or “13: Providing assistive products” may be more likely to be related to the role of a nurse associate, depending on the setting. Similarly, the “Management and Organization” practice activities which mapped to relatively few SOP documents, such as “28: Registering individuals for health services” or “35: Developing, evaluating and implementing local policies, procedures and guidelines” would likely be core responsibilities for other professions in many settings, though PA/PA-comparable professions may participate in these activities as a part of role optimization strategies in some settings.

### Economic and health workforce metrics

We found that presence or absence of certain WHO practice activities, specifically those in the “Population Health” and “Management and Organization” domains, was significantly and inversely associated with a number of economic and health workforce metrics. Most significantly, SOP documents from low- and middle-income countries reveal a much higher likelihood that the PA/PA-comparable professionals have a SOP that includes practice activities from the “Population Health” domain including: “21: Assessing community health needs,” “22: Planning and delivering community health programmes,” “23. Managing public health communication”, and “24. Developing preparedness for health emergencies and disasters, including disease outbreaks.” Likewise, within the “Management and Organization” domain, low- and middle-income countries had a higher likelihood of SOP documents that included practice activities such as “30. Providing workplace-based learning and supervision” and “34: Participating in evaluation and research” as compared to high income countries. There several potential explanations for this. For example, countries with higher health expenditures may have developed PA/PA-comparable professions to meet a narrower focus of healthcare workforce needs. As such, the practice activities in the WHO Framework in the domains of “Population Health” and “Management and Organization” may be under the responsibility of other health workers. Conversely, in countries, with lower health expenditures and smaller health workforce per capita, the PA/PA-comparable profession may have been specifically developed to meet a wider range of healthcare needs and responsibilities, or perhaps developed organically in response to local resource allocations and country-specific population health needs.

WHO advocates for role optimization to improve access and cost-effectiveness of care [11]. Role optimization thus recognizes and emphasizes that many occupational groups have overlapping roles and responsibilities to best ‘share’ the tasks in order to provide care for the many patients in need. Related to the PA/PA-comparable profession, role optimization is a strategy to address workforce gaps and remove the hierarchal implications previously described as task-shifting. In some countries there are not enough medical doctors, for example in Kenya there are 0.2 doctors per 1000 persons [12]. Healthcare expenditure also varies between countries which may influence the demand for the PA/PA-comparable workforce as they have been found to be cost-effective and have similar outcomes to doctors in their quality of care from ART administration, to caesarian sections, to routine primary care [13–18]. Thus, the broader SOP identified in low-and-middle income countries may be attributed to differences in role optimization practices as well as economic factors which support a broader scope for the PA/PA-comparable workforce in low resource settings, such as the shorter educational training, lower cost to train, and lower remuneration relative to physicians.

### Reflections on SOP documents

SOP documents varied in detail. Some were many pages while others were approximately ten lines, which may indicate variability across countries in the implied versus explicit scopes of practice governing these professions. The importance of the using the WHO framework for reference is that it clarifies what someone does, not what they know, and this literal interpretation was used to capture the data in this study. However, this highlighted to us the importance of an SOP document being unambiguous around what a health worker can or cannot do, in the interests of patient safety. Whether the WHO Framework will influence the domains of future SOP documents that are developed in the context of the framework remains to be seen. Importantly, our study does not address documents which may be better suited towards mapping competencies (e.g., competency frameworks, curricula) which may be avenues for future research.

### Strengths and limitations

Our study has several notable limitations. First, while our study sample encapsulates over 25 countries from 5 regions, our study design was a convenience sample. This may increase the likelihood that our results are impacted by selection bias. Second, SOP documents within our sample were produced from different entities from across the world (e.g., government, regulators, advocacy groups, educators) which might impact their comparability. Third, the use of both English and non-English documents through Google Translate may have created differential error in data extraction. Fourth, while we investigated several economic and workforce metrics, other metrics of medical resources which may be of interest, such as hospital bed capacity, were not included in this study, and may be avenues for future research. Additionally, the specific verbiage in the SOP documents rarely matched the exact verbiage in the WHO Framework, so the authors recognize that interpretations of what was intended by the authors of the SOP documents might differ from those of the data extractors. These limitations are counterbalanced by a number of strengths. For example, ours is the first study of an international sample of PA/PA-comparable profession SOP documents. We used a rigorous and labor-intensive data extraction methodology with multiple independent reviewers. We additionally used high-quality publicly available information on economic and healthcare expenditures in our statistical models to assess for associations between these variables and the practice activities found in the SOP documents.

## Conclusions/implications

The WHO Framework provides an effective template to catalogue and compare the responsibilities of PA/PA-comparable profession SOP documents from across the world. This study highlights that whilst the dominant responsibilities of the PA/PA-comparable profession are clustered around the “Individual Health” domain, in certain countries—usually those with a lower economic and health workforce metrics—the PA/PA-comparable professional role often additionally includes responsibilities around “Population Health” and “Management and Organization.” This exemplifies the flexibility of the PA/PA-comparable professions in meeting the different healthcare needs of different countries to include “Individual Health”, “Population Health” and “Management and Organization.”

This study utilizing the WHO Framework is the first step towards systematically understanding the variability of the PA/PA-comparable professions’ SOPs around the world. An important limitation of this approach is that SOP documents may not perfectly reflect actual granularity of clinical practice. Future research could further this understanding by examining the actual clinical practice undertaken by PA/PA-comparable professionals in different countries, as well as examine how the sociology of the professions may impact SOP development across countries. In addition, this mechanism to map PA/PA-comparable professions may be replicated to include other professions in the healthcare team. This approach could also be used to compare different occupational SOPs within a country, as well as SOPs between countries; and the evaluation in turn can then be used to inform gap analysis for training needs in the context of migration, or to better understand the health team skills mixes in different countries. The authors propose that additional information relating to the types of procedures and the level of supervision or autonomy would enable a more comprehensive comparison of SOPs, going beyond the granularity offered by the WHO framework. The findings in this study hope to influence countries to create formal SOP documents, with consideration given to the intended meaning of the verbiage used, to ensure shared understanding of the SOPs of the health workforce across professions, regulators, education institutions, patients, and communities within and between countries.

## Data Availability

Data is available from the authors upon reasonable request.
